# Comparing Molecular Methods for Early Detection and Serotyping of Enteroviruses in Throat Swabs of Pediatric Patients

**DOI:** 10.1371/journal.pone.0048269

**Published:** 2012-10-25

**Authors:** Pai-Shan Chiang, Mei-Liang Huang, Shu-Ting Luo, Tzou-Yien Lin, Kuo-Chien Tsao, Min-Shi Lee

**Affiliations:** 1 National Institutes of Infectious Diseases and Vaccinology, National Health Research Institutes, Taiwan; 2 Department of Pediatrics, Chang Gung Memorial Hospital, Taoyuan, Taiwan; 3 Graduate Institute of Clinical Medical Sciences, College of Medicine, Chang Gung University, Taoyuan, Taiwan; 4 Department of Medical Biotechnology and Laboratory Science, Chang Gung University, Taoyuan, Taiwan; 5 Department of Clinical Pathology, Chang Gung Memorial Hospital, Taoyuan, Taiwan; Johns Hopkins School of Public Health, United States of America

## Abstract

**Background:**

Enteroviruses include over 100 serotypes and usually cause self-limited infections with non-specific symptoms in children, with the exceptions of polioviruses and enterovirus 71 which frequently cause neurologic complications. Therefore, early detection and serotyping of enteroviruses are critical in clinical management and disease surveillance. Traditional methods for detection and serotyping of enteroviruses are virus isolation and immunofluorescence assay, which are time-consuming. In this study, we compare virus isolation and two molecular tests for detection and serotyping of enteroviruses in clinical samples.

**Methods:**

One hundred and ten throat swabs were collected from pediatric outpatients with enterovirus-like illnesses (hand-foot-mouth disease, herpangina, and non-specific febrile illness). Virus isolation was conducted using multiple cell lines and isolated viruses were serotyped using immunofluorescent assay. In the molecular tests, a semi-nested RT-PCR and a novel CODEHOP platform were used to detect the 5′UTR and VP1 genes of enteroviruses, respectively. Amplified nucleotides were sequenced and genotyped.

**Results:**

Among the 110 cases, 39(35%), 52(47%), and 46(42%) were tested positive with these three tests, respectively. Using the consensus results of these three tests as the gold standard, agreement of the VP1 CODEHOP test was 96%, which is higher than those of the virus isolation (89%) and the 5′-UTR test (88%). The VP1 CODEHOP test also has the best performance on serotyping confirmed with serum neutralization tests.

**Conclusions:**

The VP1 CODEHOP test performed well for detection and serotyping of enteroviruses in clinical specimens and could reduce unnecessary hospitalization cares during enterovirus seasons.

## Introduction

Human enteroviruses include over 100 serotypes and can be divided into four species using molecular typing[1]. With the exceptions of polioviruses and enterovirus 71 (EV71), which frequently cause neurological complications, human enteroviruses usually cause self-limited infections in children [2], [3]. Therefore, early detection and serotyping of enteroviruses infections are critical in clinical management and disease surveillance. The traditional standard methods for detection and serotyping of enterovirus infections are virus isolation and immunofluorescence assay (IFA), which are time-consuming and labor-intensive [4], [5]. Several clinical studies have documented that molecular diagnosis based on polymerase chain reaction (PCR) is time-saving and more sensitive than virus isolation for detection of enterovirus infections in hospitalized patients [6]–[7] [8]–[10] but few studies have been conducted in outpatients. Moreover, no study has compared molecular tests and virus isolation/IFA for serotyping of human enteroviruses using clinical specimens. Although these methods had been used to detect enteroviruses in clinical specimens including throat swabs, stool samples and cerebrospinal fluid (CSF) [5]–[7], [9]–[12], reports elucidating the comparison among the diagnostic approaches are limited.

Molecular tests for the detection of human enteroviruses in clinical specimens usually target highly conserved sites in the 5′ untranslated region (5′UTR) [13]. Due to low virus titers in clinical specimens, several reverse transcription (RT)-nested or RT-seminested PCR (RT-snPCR) have been developed to further increase its sensitivity and specificity [14]–[16]. However, serotyping of enteroviruses based on 5′UTR sequences directly amplified from clinical specimens have not been well-evaluated. In addition, enterovirus VP1 capsid gene has recently been proposed to be an ideal target for detection and serotyping of enteroviruses using the consensus degenerate hybrid oligonucleotide primer (CODEHOP) but this molecular method has not been well-evaluated in clinical specimens [17]. In this study, we compare the traditional methods (virus isolation/IFA) and these two molecular tests for detection and serotyping of human enteroviruses in throat swabs of pediatric outpatients.

## Materials and Methods

### Ethics Statement

This study was approved by the Institutional Review Board of Chang Gung Memorial Hospital (CGMH) following the Helsinki Declaration; and written informed consents were obtained from all mothers of participating infants.

### Clinical Specimens

Previous studies have shown that throat swabs are the most sensitive clinical specimens for isolation of enterovirus 71 [18]–[20]. Clinical specimens were collected from a child cohort study conducted in northern Taiwan [21]–[23]. In the cohort study, sera were obtained from participating children in the following schedule: neonates at birth (cord blood), and children at 6, 12, 24, and 36 months of age. If the participating children developed suspected enterovirus illnesses (herpangina, hand-foot-mouth disease, and non-specific febrile illness), throat swabs and sera were collected from these participating children. In 2008–09, 153 suspected cases were detected in the study cohort and 118 of them provided throat swabs. Among the 118 cases providing throat swabs, 110 had completed virus isolation and molecular tests and 94 of them also provided paired sera samples.

### Virus Isolation and Serotyping with Immunofluorescent Assay (IFA)

Clinical specimens were inoculated onto four commercial cell lines (Hep2, MK2, MRC-5, and RD) obtained from ATCC (Manassas, Virginia, USA), and cells with cytopathic effect were harvested for IFA using antibodies which can detect respiratory syncytial virus, herpes simplex virus, influenza virus, parainfluenza virus, cytomegalovirus and enterovirus. If the specimens were positive for enterovirus testing, serotype-specific monoclonal antibodies were further employed to identify serotypes of enteroviruses. The type-specific monoclonal antibodies covered 23 serotypes, including Polio 1–3; coxsackievirus A2, A4, A5, A6, A9, A10, A16, A24, B1-6; Echovirus 4, 6, 9, 11, 30; and EV71 [5].

### Viral RNA Extraction and Semi-Nested RT-PCR

Viral RNA was extracted from the clinical specimens using a QIAamp Mini Viral RNA Extraction Kit (Qiagen, Germany). A RT-PCR procedure for each amplification was performed in a 50 µl reaction mixture with 5 µl of extracted RNA template and the Access Ouick™ RT-PCR system (Promega, Wisconsin, USA). The highly conserved 5′UTR of the enteroviruses was chosen as the target for the synthesis of a 440-bp cDNA with primers EV-F1 and EV-R1 (Table 1). The cycling conditions were as follows: 45 min at 45°C for the RT step, 2 min at 95°C for the initial denature step, 40 cycles for 30 s at 95°C for denature, 30 s at 52°C for annealing, 1 min at 72°C for extension, and a final extension at 72°C for 10 min. Negative controls (sterile water) were included in each amplification series. To improve sensitivity and specificity for identifying enterovirus, a semi-nested PCR system was applied (Table 1) [24]. Semi-nested PCR was performed with 5 µl of each RT-PCR product added to a new PCR tube with a solution containing 10x PCR buffer 5.0 µl (20 mmol/l MgCl_2_), dNTP mix 2.0 µl (10 mmol/l of each dNTP), EV-F1/EV-R1N 1.0 µl (10 µmol/l for seminested PCR), and VioTaq DNA polymerase 0.5 µl (5 units/µl), (Viogene, Taiwan). Sterile water was added to a final volume of 50 µl. The following temperature program was used: 40 cycles consisting of 95°C for 30 s, 56°C for 30 s, 72°C for 1 min and a final extension at 72°C for 10 min. The semi-nested primers, EV-F1 and EV-R1N, yielded a 400-bp amplicon. Negative controls (sterile water) were included in each amplification series. One EV71 and one CA16 isolate were used as positive controls of RT-PCR for enterovirus detection.

**Table 1:** Primers used for 5′UTR RT-PCR and VP1 CODEHOP tests.

Primer	Position^a^	Sequence (5′-3′)^b^	Target region	Polarity
EV-F1	172–192	CAAGCAYWTCTGTWYCCCCGG	5′ UTR	sense
EV-R1	588–607	ATTGTCACCATAAGCAGYCR	5′ UTR	antisense
EV-R1N	552–568	CACGGACACCCAAAGTA	5′ UTR	antisense
AN32	2956–2949	GTYTGCCA	VP1	antisense
AN33	2956–2949	GAYTGCCA	VP1	antisense
AN34	3058–3051	CCRTCRTA	VP1	antisense
AN35	2956–2949	RCTYTGCCA	VP1	antisense
224	1927–1946	GCIATGYTIGGIACICAYRT	VP3	sense
222	2916–2898	CICCIGGIGGIAYRWACAT	VP1	antisense
AN89	2570–2595	CCAGCACTGACAGCAGYNGARAYNGG	VP1	sense
AN88	3166–3146	TACTGGACCACCTGGNGGNAYRWACAT	VP1	antisense

a The positions of primers are those relative to the genome of EV71 BrCr (GenBank accession number U22521).^b^ Degenerate bases followed nucleotide IUB codes.

### PCR Amplification and Serotyping Using CODEHOP

EV VP1 gene sequences were amplified by a recently described CODEHOP protocol leading to product of 350∼400 bp [17], [25]. In brief, virus RNA was extracted from clinical specimens using a QIAamp Mini Viral RNA Extraction Kit (Qiagen, Germany) and cDNA was generated using four different primers (AN32-35). The cDNA was then used in the first PCR with primers AN88 and AN89. Product of the first PCR was added to a second PCR with primers 222 and 224 for nested amplification (Table 1).

### Sequence Analysis

The amplified DNA was sequenced using the ABI 3730 XL DNA Analyzer (Applied Biosystem Inc., Foster City, CA). Nucleotide sequences of 5′UTR or VP1 were checked against the NCBI database by the BLAST search to find the enterovirus serotype with the highest identity. Alignment of the nucleotide sequences of the enterovirus isolates was performed using BioEdit Sequence Alignment Editor software v7.08 (Tom Hall, North Carolina State University, Carolina, USA). A phylogenetic dendrogram was constructed using the neighbor-joining method of the MEGA program v4.0 (Arizona State University, AZ, USA). Nucleotide sequences analyzed in this study have been submitted to GenBank (accession numbers JN896765–JN896862).

### Serologic Assay

Serum neutralizing antibody test has been widely used for serotyping of enterovirus isolates. However, it is not suitable for early diagnosis of enterovirus infections due to requirement of collecting paired sera and complexity of preparing virus stocks. Therefore, we only used serum neutralizing antibody test to verify serotypes of enterovirus infections when IFA and molecular tests had disagreed results. Serotypes used for serum neutralization tests included EV71, coxsackievirus A2, A4, A5, A6 and A10. Laboratory methods for measuring serum neutralizing antibody titers followed standard protocols [26]. Twofold serially diluted sera and virus working solution containing 100 TCID_50_ of enterovirus were mixed on 96-well microplates and incubated with rhabdomyosarcoma cells. A cytopathic effect was observed in a monitor linked with an inverted microscope after an incubation period of 4 to 5 days. The neutralization titers were read as the highest dilution that could result in a 50% reduction in the cytopathic effect. Each test sample was run simultaneously with cell control, serum control, and virus back titration. The starting dilution was 1∶8 and the cutoff level of seropositivity was set at 8.

### Statistical Analysis

Since gold standard is not available for detection of human enteroviruses in clinical samples, consensus result of the three tests (virus isolation and two molecular tests) was used as the gold standard to evaluate individual performance of these three tests by calculating sensitivity, specificity, positive prediction value, negative prediction value, and agreement [27], [28]. The statistical significance in the agreement rates of different tests were tested by the *X^2^* test and Fisher's exact test as appropriate. All statistical analyses were performed using Epi-Info (CDC, Atlanta, GA) or SAS (SAS Institute, Cary, NC).

## Results

### Detection of Enteroviruses in Throat Swabs

Between January 2008 and December 2009, throat swabs were collected from 110 children diagnosed with HFMD (7 cases), herpangina (93 cases), and non-specific febrile illness (10 cases). Among them, 39 (35%), 52 (47%), and 46 (42%) were tested positive by the virus isolation/IFA, the 5′UTR RT-PCR, and the VP1 CODEHOP tests, respectively. Overall, results of these 110 samples tested with these three methods have eight combinations, including 30 were tested positive by all of these three tests, 17 were positive by two of these three tests, 13 were positive by only one of these three tests, and 50 were negative with all of these three tests (Table 2). Since no gold standard method has been established for detection of enteroviruses in throat swabs, it would be reasonable to use consensus result of these three tests as the gold standard for evaluating performance of each individual test. Using the consensus result of these three tests as the gold standard, positive prediction value (PPV), negative prediction value (NPV) and agreement of these three tests were 95%, 86% and 89% for the virus isolation/IFA test, 83%, 93% and 88% for the 5′-UTR test, and 96%, 95%, and 96% for the VP1 CODEHOP test, respectively (Table 3). Overall, agreement of the VP1 CODEHOP test was higher than that of the virus isolation and 5′UTR tests (P = 0.05, Fisher's exact test).

**Table 2:** Detection of enteroviruses in 110 throat swabs using three tests.

Virus isolation	5′UTR RT-PCR	VP1 CODEHOP	No. of tests
+	+	+	30 (23, 7)^a^
+	+	−	3 (1, 2)^a^
+	−	+	4 (4,0)^a^
−	+	+	10 (8,2)^a^
+	−	−	2
−	+	−	9
−	−	+	2
−	−	−	50

a numbers in parenthesis indicate agreed and disagreed serotyping.

**Table 3:** Evaluation of three diagnostic methods for detection of enteroviruses in 110 clinical specimens.

Consensus result*	Virus isolation		5′UTR RT-PCR		VP1 CODEHOP	
	+	−	+	−	+	−
+	37	10	43	4	44	3
−	2	61	9	54	2	61
Sensitivity	0.787		0.915		0.936	
Specificity	0.968		0.855		0.968	
PPV	0.949		0.827		0.957	
NPV	0.859		0.930		0.953	
Agreement	0.891		0.881		0.955	

PPV: positive prediction value; NPV: negative prediction value. * Consensus results were based on the majority results of the three methods evaluated.

### Serotyping of Enteroviruses in Clinical Specimens

Among the 30 cases tested positive by all of these three tests, 23 had agreed serotypes and 7 had disagreed serotypes. Except one case who did not provide post-infection serum, 6 of the 7 cases with disagreed serotypes were further verified to be consistent with the virus isolation and VP1 CODEHOP tests by serum neutralization test using paired sera collected before and after infections (Table 4). Among the 17 cases tested positive by two of these three tests, 13 had agreed serotypes and 4 had disagreed serotypes. Among these 4 cases with disagreed serotypes, 2 cases were serologically verified to be consistent with the virus isolation (ID#98050) or the VP1 CODEHOP tests (ID#97009), respectively and the remaining 2 cases (ID#97035 and ID#98055) could not be verified (Table 5). There were 13 cases tested positive by only one of the three tests and they were further serologically verified (Table 5). Among 2 cases (ID#97028 and ID#97065) tested positive only by the virus isolation/IFA, one was serologically confirmed and one did not provide post-infection serum. Among 9 cases tested positive only by the 5′-UTR test, seven were confirmed serologically to be false positive and two did not provide post-infection sera. Among two cases (ID#98019 and ID#98044) tested positive only by the VP1 CODEHOP test, both were serologically confirmed . Overall, the serotyping results further confirmed superiority of the VP1 CODEHOP to the 5′-UTR test. Table 6 showed the distribution of the top 5 enterovirus serotypes detected in Taiwan, 2008-09. The VP1 CODEHOP test could detect much more EV71 infections than the virus isolation (9 cases vs. 4 cases) and eight of these nine cases developed seroconversion against EV71 except one case who did not provide post-infection serum, which further confirmed accuracy of the VP1 CODEHOP test.

**Table 4:** Serologic tests in patients who have disagreed serotypes by three compared tests.

Swab ID	Virus isolation	5′UTR RT-PCR	VP1 CODEHOP	Symptom	Neutralizing antibody seroconversion (serotype)
97036	EV71	CA2	EV71	Herpangina	Yes (EV71)
97041	CB4	CA9	CB4	Herpangina	Not available
98020	CA4	CA3	CA4	Herpangina	Yes (CA4)
98034	CA10	CA6	CA10	Herpangina	Yes (CA10)
98048	CA4	CA3	CA4	Herpangina	Yes (CA4)
98061	CA4	CA3	CA4	Herpangina	Yes (CA4)
98062	CA4	CA3	CA4	Herpangina	Yes (CA4)

**Table 5:** Serologic verification in patients with enterovirus detection by one or two of the three compared tests.

ID	Virus isolation	5′UTR RT-PCR	VP1 CODEHOP	Symptom	Neutralizing antibody seroconversion (serotype)
Patients with enterovirus detection by two of the three compared tests					
97035	CA2	CA5	negative	Herpangina	Yes (CA2), Yes (CA5)
98050	CA10	CA6	negative	Herpangina	Yes (CA10), No (CA6)
97009	negative	CA5	EV71	HFMD	No (CA5), Yes (EV71)
98055	negative	CA16	EV71	Herpangina	No post-infection serum
Patients with enterovirus detection by one of the three compared tests					
97028	CA2	negative	negative	Herpangina	Yes (CA2)
97065	CA10	negative	negative	Herpangina	No post-infection serum
97006	negative	CA6	negative	Herpangina	No post-infection serum
97007	Adenovirus	CA10	negative	Herpangina	No post-infection serum
97008	Parainfluenza-3	EV71	negative	Herpangina	No (EV71)
97014	Cytomegalovirus	CA5	negative	Fever, sore throat	No (CA5)
97045	HSV-1	EV71	negative	Herpangina	No (EV71)
97051	negative	EV71	negative	Fever, rash	No (EV71)
98031	HSV-1	CA5	negative	Herpangina	No (CA5)
98037	RSV	CA5	negative	Herpangina	No (CA5)
98047	Adenovirus	CA5	negative	Herpangina	No (CA5)
98019	Cytomegalovirus	negative	CA5	Herpangina	Yes (CA5)
98044	Cytomegalovirus	negative	CA5	Herpangina	Yes (CA5)

HFMD: hand-foot-mouth disease

**Table 6:** Top five serotypes of human enteroviruses detected by three different tests in 110 clinical specimens.

Test	Top 1	Top 2	Top 3	Top 4	Top 5
Virus isolation	CA6 (8%, 9)	CA10 (8%, 9)	CA2 (6%, 7)	CA4 (4%, 4)	EV71 (4%, 4)
5′-UTR RT-PCR	CA6 (9%, 10)	CA2 (8%, 9)	CA5 (7%, 8)	CA10 (7%, 8)	EV71 (7%, 8)
VP1 CODEHOP	CA6 (9%, 10)	EV71 (8%, 9)	CA10 (7%, 8)	CA2 (6%, 7)	CA4 (4%, 4)
Consensus a	CA6 (9%, 10)	CA2 (8%, 9)	CA10 (8%, 9)	EV71 (7%, 8)	CA4 (4%, 4)

a Based on the consensus results of these three tests.

## Discussion

Human enteroviruses have more than 100 serotypes which make laboratory diagnosis very challenging. Traditional methods based on virus isolation and IFA are time-consuming and labor-intensive and could not detect new serotypes before antisera are available. In this study, we compare virus isolation/IFA and two molecular tests targeting 5′UTR and VP1 genes for detection and serotyping of human enteroviruses in clinical specimens of pediatric outpatients. Overall, the VP1 CODEHOP test has the best performance on detection and serotyping, which confirmed preliminary finding from a previous study which only tested 7 clinical specimens [17].

Molecular tests are more sensitive than virus isolation for detection of enteroviruses but may have more false positive reactions which can be differentiated by determining seroconversion of serotype-specific neutralizing antibody. Most studies did not collect paired sera to measure serotype-specific serum neutralizing antibody in outpatient studies [7], [8], [29]. In our study, paired sera have been collected to verify the validity of the molecular tests. It is not surprising to found that detection rate of the virus isolation/IFA was lower than those of the 5′UTR RT-PCR and VP1 CODEHOP tests (35%, 47%, and 42%, respectively). After verification with serotype-specific serum neutralization test, we found that the 5′UTR RT-PCR test was more likely to have false positive reactions (Table 4). In addition, the 5′UTR-PCR test was also more likely to have false serotyping.

There are several possible reasons why the 5′UTR-PCR test was more likely to have the false positive reaction and mismatched serotyping than the VP1 CODEHOP. First, the 5′UTR gene is more conservative than the VP1 gene among viruses of the Enterovirus genus which includes at least 10 species (4 human enteroviruses, 3 human rhinoviruses, and 3 animal enteroviruses) [30] . Second, phylogenetic analysis using the VP1 gene sequences is highly correlated to serotyping using serum neutralization tests in enteroviruses [31]. Third, gene recombinations between enteroviruses have been detected in the 5′UTR region but not in the VP1 region [32], [33]. At the moment, standard laboratory methods for enterovirus surveillance have not been established, which make international comparison impossible. An international human enterovirus surveillance network would be desirable to establish standard molecular and serological methods for laboratory diagnosis of human enterovirus infections and understand their disease burden.

Most of enterovirus infections are self-limited and do not require hospitalization cares. However, EV71 infections in young children frequently cause complications and could progress quickly. Therefore, Taiwanese parents frequently requests hospitalization cares if their children develop enterovirus-like symptoms (HFMD, herpangina and febrile illness) during EV71 epidemics. In a previous study, only 18.9% of enterovirus inpatients were confirmed to have EV71 infections during the 2008 EV71 epidemics in Taiwan [22]. Rapid tests differentiating EV71 infections from other enterovirus infections are urgently needed to reduce unnecessary hospitalization cares. The virus isolation/IFA test requires 5–14 days to complete detection and serotyping, which is useful to virus surveillance but not clinical management. In contrast, the VP1 CODEHOP test could finish detection within 24 hours and requires another 24 hours for serotyping. Therefore, the VP1 CODEHOP test could be used to reduce unnecessary hospitalization cares during EV71 epidemics. In addition, the VP1 CODEHOP test could more precisely estimate disease burden of EV71 infections than the virus isolation in epidemiological studies. Moreover, about 15∼30% of enterovirus isolates could not be serotyped annually using IFA in Taiwan [5]. The VP1 CODEHOP platform could potentially reduce the number of untypable enterovirus isolates and detect new enterovirus serotypes [1], [34].

## References

[1] Yozwiak NL, Skewes-Cox P, Gordon A, Saborio S, Kuan G, et al (2010) Human enterovirus 109: a novel interspecies recombinant enterovirus isolated from a case of acute pediatric respiratory illness in Nicaragua. J Virol 84: 9047–9058. 20592079 10.1128/JVI.00698-10 PMC2937614

[2] Lee MS, Chang LY (2010) Development of enterovirus 71 vaccines. Expert Rev Vaccines 9: 149–156. 20109026 10.1586/erv.09.152

[3] Solomon T, Lewthwaite P, Perera D, Cardosa MJ, McMinn P, et al (2010) Virology, epidemiology, pathogenesis, and control of enterovirus 71. Lancet Infect Dis 10: 778–790. 20961813 10.1016/S1473-3099(10)70194-8

[4] Lin TL, Li YS, Huang CW, Hsu CC, Wu HS, et al (2008) Rapid and highly sensitive coxsackievirus a indirect immunofluorescence assay typing kit for enterovirus serotyping. J Clin Microbiol 46: 785–788. 18032614 10.1128/JCM.01114-07 PMC2238120

[5] Tsao KC, Huang CG, Huang YL, Chen FC, Huang PN, et al (2010) Epidemiologic features and virus isolation of enteroviruses in Northern Taiwan during 2000–2008. J Virol Methods 165: 330–332. 20214926 10.1016/j.jviromet.2010.03.001

[6] Robinson CC, Willis M, Meagher A, Gieseker KE, Rotbart H, et al (2002) Impact of rapid polymerase chain reaction results on management of pediatric patients with enteroviral meningitis. Pediatr Infect Dis J 21: 283–286. 12075757 10.1097/00006454-200204000-00005

[7] Singh S, Chow VT, Phoon MC, Chan KP, Poh CL (2002) Direct detection of enterovirus 71 (EV71) in clinical specimens from a hand, foot, and mouth disease outbreak in Singapore by reverse transcription-PCR with universal enterovirus and EV71-specific primers. J Clin Microbiol 40: 2823–2827. 12149336 10.1128/JCM.40.8.2823-2827.2002 PMC120643

[8] Tsao LY, Lin CY, Yu YY, Wang BT (2006) Microchip, reverse transcription-polymerase chain reaction and culture methods to detect enterovirus infection in pediatric patients. Pediatr Int 48: 5–10. 16490062 10.1111/j.1442-200X.2006.02157.x

[9] Vuorinen T, Vainionpaa R, Hyypia T (2003) Five years' experience of reverse-transcriptase polymerase chain reaction in daily diagnosis of enterovirus and rhinovirus infections. Clin Infect Dis 37: 452–455. 12884172 10.1086/376635

[10] Wu Y, Yeo A, Phoon MC, Tan EL, Poh CL, et al (2010) The largest outbreak of hand; foot and mouth disease in Singapore in 2008: the role of enterovirus 71 and coxsackievirus A strains. Int J Infect Dis 14: e1076–1081. 20952237 10.1016/j.ijid.2010.07.006

[11] Huang YP, Lin TL, Hsu LC, Chen YJ, Tseng YH, et al (2010) Genetic diversity and C2-like subgenogroup strains of enterovirus 71, Taiwan, 2008. Virol J 7: 277. 20959020 10.1186/1743-422X-7-277 PMC2975644

[12] Tryfonos C, Richter J, Koptides D, Yiangou M, Christodoulou CG (2011) Molecular typing and epidemiology of enteroviruses in Cyprus, 2003–2007. J Med Microbiol 60: 1433–1440. 21596905 10.1099/jmm.0.029892-0

[13] Romero JR (1999) Reverse-transcription polymerase chain reaction detection of the enteroviruses. Arch Pathol Lab Med 123: 1161–1169. 10583920 10.5858/1999-123-1161-RTPCRD

[14] Casas I, Palacios GF, Trallero G, Cisterna D, Freire MC, et al (2001) Molecular characterization of human enteroviruses in clinical samples: comparison between VP2, VP1, and RNA polymerase regions using RT nested PCR assays and direct sequencing of products. J Med Virol 65: 138–148. 11505456

[15] Nix WA, Berger MM, Oberste MS, Brooks BR, McKenna-Yasek DM, et al (2004) Failure to detect enterovirus in the spinal cord of ALS patients using a sensitive RT-PCR method. Neurology 62: 1372–1377. 15111676 10.1212/01.wnl.0000123258.86752.51

[16] Thoelen I, Lemey P, Van Der Donck I, Beuselinck K, Lindberg AM, et al (2003) Molecular typing and epidemiology of enteroviruses identified from an outbreak of aseptic meningitis in Belgium during the summer of 2000. J Med Virol 70: 420–429. 12767006 10.1002/jmv.10412

[17] Nix WA, Oberste MS, Pallansch MA (2006) Sensitive, seminested PCR amplification of VP1 sequences for direct identification of all enterovirus serotypes from original clinical specimens. J Clin Microbiol 44: 2698–2704. 16891480 10.1128/JCM.00542-06 PMC1594621

[18] Chang LY, Lin TY, Huang YC, Tsao KC, Shih SR, et al (1999) Comparison of enterovirus 71 and coxsackie-virus A16 clinical illnesses during the Taiwan enterovirus epidemic, 1998. Pediatr Infect Dis J 18: 1092–1096. 10608631 10.1097/00006454-199912000-00013

[19] Chang LY, Hsia SH, Wu CT, Huang YC, Lin KL, et al (2004) Outcome of enterovirus 71 infections with or without stage-based management: 1998 to 2002. Pediatr Infect Dis J 23: 327–332. 15071287 10.1097/00006454-200404000-00010

[20] Ooi MH, Solomon T, Podin Y, Mohan A, Akin W, et al (2007) Evaluation of different clinical sample types in diagnosis of human enterovirus 71-associated hand-foot-and-mouth disease. J Clin Microbiol 45: 1858–1866. 17446325 10.1128/JCM.01394-06 PMC1933032

[21] Luo ST, Chiang PS, Chao AS, Liou GY, Lin R, et al (2009) Enterovirus 71 maternal antibodies in infants, Taiwan. Emerg Infect Dis 15: 581–584. 19331737 10.3201/1504.081550 PMC2671432

[22] Lee MS, Lin TY, Chiang PS, Li WC, Luo ST, et al (2010) An investigation of epidemic enterovirus 71 infection in Taiwan, 2008: clinical, virologic, and serologic features. Pediatr Infect Dis J 29: 1030–1034. 20543760 10.1097/INF.0b013e3181e52945

[23] Lee MS, Chiang PS, Luo ST, Huang ML, Liou GY, et al (2012) Incidence Rates of Enterovirus 71 Infections in Young Children during a Nationwide Epidemic in Taiwan, 2008-09. PLoS Negl Trop Dis 6: e1476. 22348156 10.1371/journal.pntd.0001476 PMC3279337

[24] Hafliger D, Gilgen M, Luthy J, Hubner P (1997) Seminested RT-PCR systems for small round structured viruses and detection of enteric viruses in seafood. Int J Food Microbiol 37: 27–36. 9237119 10.1016/s0168-1605(97)00041-x

[25] Ortner B, Huang CW, Schmid D, Mutz I, Wewalka G, et al (2009) Epidemiology of enterovirus types causing neurological disease in Austria 1999–2007: detection of clusters of echovirus 30 and enterovirus 71 and analysis of prevalent genotypes. J Med Virol 81: 317–324. 19107980 10.1002/jmv.21374

[26] Huang ML, Chiang PS, Luo ST, Liou GY, Lee MS (2010) Development of a high-throughput assay for measuring serum neutralizing antibody against enterovirus 71. J Virol Methods 165: 42–45. 20036286 10.1016/j.jviromet.2009.12.015

[27] Lee MS, Cohen B, Hand J, Nokes DJ (1999) A simplified and standardized neutralization enzyme immunoassay for the quantification of measles neutralizing antibody. J Virol Methods 78: 209–217. 10204711 10.1016/s0166-0934(98)00178-5

[28] Lee MS, Chen JS (2004) Predicting antigenic variants of influenza A/H3N2 viruses. Emerg Infect Dis 10: 1385–1390. 15496238 10.3201/eid1008.040107 PMC3320420

[29] Zhang Y, Tan XJ, Wang HY, Yan DM, Zhu SL, et al (2009) An outbreak of hand, foot, and mouth disease associated with subgenotype C4 of human enterovirus 71 in Shandong, China. J Clin Virol 44: 262–267. 19269888 10.1016/j.jcv.2009.02.002

[30] Knowles NJ, Hovi T, Hyypia T, King AMQ, Lindberg AM, et al.. (2012) Family Picornaviridae. Virus Taxonomy: Ninth Report of the International Committee on Taxonomy of Viruses. London: Elsevier Inc. pp. 855–880.

[31] Pallansch M, Roos RP (2007) Enteroviruses: polioviruses, coxsackieviruses, echoviruses, and newer enteroviruses. In: Knipe DM, Howley PM, editors. Fields Virology. 5th ed. New York: Raven Press. pp. 839–893.

[32] Chan YF, AbuBaker S (2004) Recombinant human enterovirus 71 in hand, foot and mouth disease patients. Emerg Infect Dis 10: 1468–1470. 15496251 10.3201/eid1008.040059 PMC3320397

[33] Yoke-Fun C, AbuBakar S (2006) Phylogenetic evidence for inter-typic recombination in the emergence of human enterovirus 71 subgenotypes. BMC Microbiol 6: 74. 16939656 10.1186/1471-2180-6-74 PMC1569848

[34] Ryu WS, Kang B, Hong J, Hwang S, Kim J, et al (2010) Clinical and etiological characteristics of enterovirus 71-related diseases during a recent 2-year period in Korea. J Clin Microbiol 48: 2490–2494. 20463159 10.1128/JCM.02369-09 PMC2897491

